# Prolonged Paroxysmal Sympathetic Storming Associated with Spontaneous Subarachnoid Hemorrhage

**DOI:** 10.1155/2013/358182

**Published:** 2013-02-11

**Authors:** Yan Liu, Suneil Jolly, Krishna Pokala

**Affiliations:** ^1^Department of Internal Medicine, University of Texas Southwestern Medical School Residency Programs at Austin, University Medical Center Brackenridge, 601 East 15th Street, Austin, TX 78701, USA; ^2^Transitional Program, University of Texas Southwestern Medical School Residency Programs at Austin, University Medical Center Brackenridge, Austin, TX 78701, USA; ^3^Department of Neurology, University of Texas Southwestern Medical School Residency Programs at Austin, University Medical Center Brackenridge, Austin, TX 78701, USA

## Abstract

Paroxysmal sympathetic storming (PSS) is a rare disorder characterized by acute onset of nonstimulated tachycardia, hypertension, tachypnea, hyperthermia, external posturing, and diaphoresis. It is most frequently associated with severe traumatic brain injuries and has been reported in intracranial tumors, hydrocephalous, severe hypoxic brain injury, and intracerebral hemorrhage. Although excessive release of catecholamine and therefore increased sympathetic activities have been reported in subarachnoid hemorrhage (SAH), there is no descriptive report of PSS primarily caused by spontaneous SAH up to date. Here, we report a case of prolonged PSS in a patient with spontaneous subarachnoid hemorrhage and consequent vasospasm. The sympathetic storming started shortly after patient was rewarmed from hypothermia protocol and symptoms responded to Labetalol, but intermittent recurrence did not resolve until 3 weeks later with treatment involving Midazolam, Fentanyl, Dexmedetomidine, Propofol, Bromocriptine, and minimizing frequency of neurological and vital checks. In conclusion, prolonged sympathetic storming can also be caused by spontaneous SAH. In this case, vasospasm might be a precipitating factor. Paralytics and hypothermia could mask the manifestations of PSS. The treatment of the refractory case will need both timely adjustment of medications and minimization of exogenous stressors or stimuli.

## 1. Introduction

Paroxysmal sympathetic storming, also known as paroxysmal autonomic instability with dystonia [1] and diencephalic seizures [2], has been defined as the presence of tachycardia, hypertension, tachypnea, hyperthermia, dystonia, posturing, and diaphoresis with a minimum of 1 cycle per day in a patient with severe brain injury or other equivalent conditions [1].

The pathophysiology of sympathetic storming has been refined since it was originally described in 1929, and it was initially thought to be resulted from epileptic discharges from thalamic nuclei [3]. However, based on EEG studies [4] and the current model, it is recognized that the interruption of autonomic pathways results in an imbalance between sympathetic and parasympathetic nervous systems, which leads to PSS [2]. There is no clear pattern of injury that increases the likelihood of sympathetic hyperactivity. However, it is more common in patients with diffuse axonal injury [2, 5].

Sympathetic storming occurs most frequently in patients with traumatic brain injury [6–9]. However, this unique phenomenon has also been associated with hypoxic injury [5], brain tumors [2, 10], and hydrocephalous [2, 11]. Although excessive release of catecholamine and increased sympathetic activities resulting in cardiac and pulmonary manifestations have been well reported in subarachnoid hemorrhage [12–14], for example, myocardial infarction and hypertension, there was no report with specific description of paroxysmal sympathetic storming in spontaneous SAH found after extensive literature search. Here, we report a case in which a patient developed prolonged sympathetic storming after a spontaneous subarachnoid hemorrhage secondary to a posterior communicating aneurysm rupture. The prevention and management of PSS in this setting is also discussed.

## 2. Case Report

**Figure 1:**
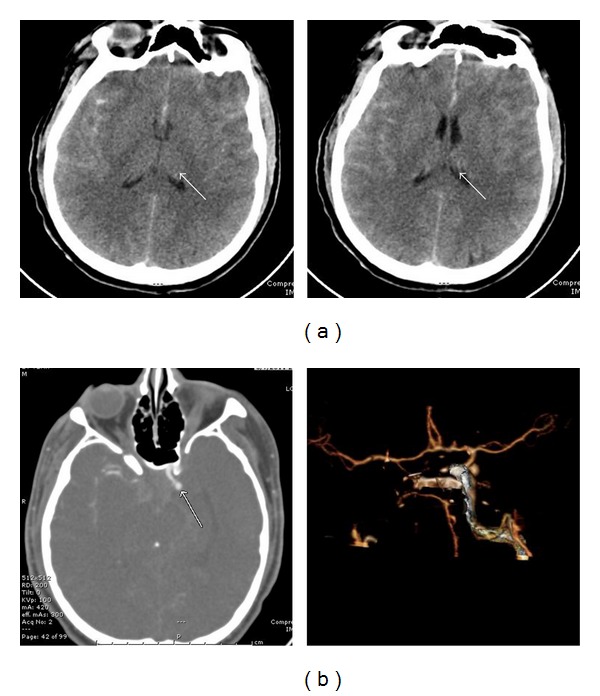
Imaging studies showing spontaneous subarachnoid hemorrhage. (a) CT head without contrast showed subarachnoid hemorrhage with intraventricular involvement. (b) CT angiogram of brain demonstrated a 4-5 mm saccular left posterior communicating artery aneurysm.

**Figure 2:**
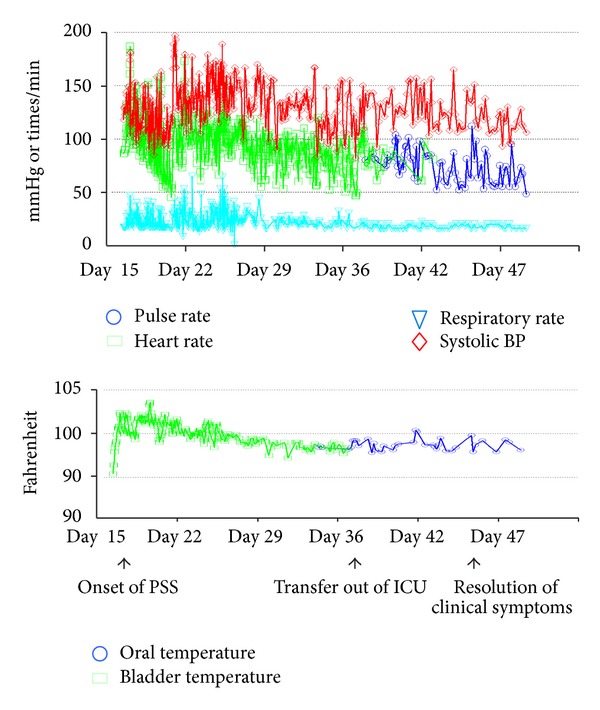
The evolvement of vital sign changes during patient's hospitalization. The extent of changes in heart rate/pulse, temperature, respiratory rate, and systolic blood pressure during PSS episodes is shown throughout hospital course.

A 27-year-old Hispanic man with no known medical history arrived to the hospital via EMS after complaining of a headache one day prior to arrival. He had lost his consciousness and collapsed before being intubated and admitted to ICU. A CT head without contrast showed a subarachnoid hemorrhage and a CT angiogram of brain demonstrated a 4-5 mm saccular left posterior communicating artery aneurysm (Figure 1). An urgent ventriculostomy was performed as well as craniotomy with posterior aneurysmal clipping. Despite Nimodipine for vasospasm prevention, the patient developed episodes of vessel spasms and increased intracranial pressure (ICP). Due to these complications, the patient was placed on a hypothermic protocol with paralytics. Mannitol and 3% sodium chloride were started for ICP elevation. Levetiracetam was added for seizure prophylaxis. After his ICP was normalized, the patient was weaned off all paralytics and rewarmed from the hypothermia protocol on day 16 of hospitalization. Overnight on day 16 of hospitalization, however, the patient developed acute onset of episodic tachycardia up to 200, hypertension with SBP up to 220, increased respiratory rate into the mid 30s, and hyperthermia with a temperature of 101 F. Meanwhile, he had severe diaphoresis, tremor, and spontaneous extensor posturing. All of this occurred with a normal ICP and no change in gag reflex or pupil size. The clinical episodes were consistent with sympathetic storming. He was given Labetalol IV 10 mg twice, and his tachycardia, hypertension, posturing, and tremor quickly resolved within several minutes while his hyperthermia and diaphoresis improved gradually. This rapid response to Labetalol treatment confirmed a diagnosis of sympathetic storm. There was no evidence indicating possible acute onset infection, hyperthyroidism, pheochromocytoma, or hypercortisolism. Despite this initial treatment, similar episodes recurred with frequency of 3–6 episodes/day with duration range from 30 minutes to 2 hours, each episode was accompanied by significant changes of vital signs in cycles (Figure 2). In addition to Labetalol and Bromocriptine as needed, Dexmedetomidine, Fentanyl, and Midazolam were also used in an attempt to control these symptoms. However, the patient continued to exhibit episodic storming symptoms during his ICU stay and a tracheostomy and PEG tube had to be placed. He was subsequently started on scheduled Propranolol 10 mg BID and Bromocriptine 5 mg TID in addition to Labetalol as needed before being transferred to the intermediate care unit with less frequent interruption/stimuli by neurologic and vital checks.

His symptoms subsequently improved and the relapses of PSS steadily dissipated (Figure 2). His storming symptoms completely resolved on day 46 of his hospitalization. Although not fully oriented, the patient resumed normal spontaneous movement of extremity and eye opening with the ability of simple command following. His Bromocriptine and Propranolol were then switched to PRN and later discontinued. He was then discharged to nursing facility with 24-hour care.

## 3. Discussion

Although the general clinical presentation of paroxysmal sympathetic storming has been well recognized for nearly a century [3], the syndrome has not been described in a nontraumatic subarachnoid hemorrhage. Most well recognized cases of sympathetic storming have been identified in patients who have suffered traumatic brain injury [6–9], brain tumors [2, 10], aqueductal stenosis [15], or cardiac arrest [16]. Subarachnoid hemorrhage is associated with significant catecholamine elevation and marked sympathetic activation [17, 18], which has been linked to cardiopulmonary complication other than PSS [12–14, 19, 20], such as neurogenic stress cardiomyopathy, arrhythmias, neurogenic pulmonary edema, and neurogenic myocardial injury [21–24]. While numerous studies have described the above conditions in SAH, in our extensive electronic literature search for English-language articles on neurological and cardiopulmonary complications of SAH up to date, no original article was found reporting the direct association of paroxysmal sympathetic storming (or its alias) with spontaneous SAH. This paper presents the description of PSS caused by spontaneous SAH with a prolonged hospital course that involved comprehensive management of this syndrome.

It is essential to distinguish PSS from sympathetic activation-induced cardiopulmonary effects, as their manifestation, mechanism, and treatment are different. While hypertension and cardiac injury are relative common complications of SAH and are a result of catecholamine elevation [19, 21–24], PSS is rare and manifested as episodic tachycardia, hypertension, tachypnea, hyperthermia, dystonia, posturing, and diaphoresis in cycles. Instead of simple sympathetic activation, PSS is thought to be caused by the imbalance between sympathetic and parasympathetic nervous systems [2]. The mechanism contributing to its paroxysmal nature is not clear although certain external noxious stimuli may act as triggering factors. As in this case, the initial vasospasm and transiently increased ICP may be triggering factor and the signs of PSS might have been masked by hypothermia initially as storming symptoms started right after rewarming, days after his ICP was normalized. In this case, although PSS symptoms promptly responded to nonselective *β*-blocker Labetalol upon onset, the episodic relapses did not dissipate till scheduled Propranolol and Bromocriptine were started in addition to sedative agents and the external stimuli including vital and neurologic checks were minimized to medical necessity. Therefore, we think that the scheduled Bromocriptine and Propranolol are much more effective in inducing complete resolution of PSS symptoms.

This patient's sympathetic storming did not start until he was weaned from the hypothermia protocol, this frequent association between PSS and rewarming as well as weaning of sedatives and paralytics has been well reported [16]. This raised question for potential prophylaxis of PSS in these high-risk patients with medication that has minimal effect on hemodynamics such as scheduled Bromocriptine. The potential prevention of PSS in critically ill patients may significantly reduce related complications, increase their survival, and shorten their ICU stay.

In conclusion, we report a case of prolonged paroxysmal sympathetic storming as a rare complication of spontaneous SAH, which is related to, but distinct from, commonly reported sympathetic activation secondary to SAH. The signs and symptoms of PSS may be precipitated by external stimuli and stressors, but can be masked by paralytics and hypothermia. The treatment of the refractory case will need both timely adjustment of antisympathetic medications and minimization of exogenous stressors/stimuli. Future studies are needed to determine the benefit of PSS prophylaxis with scheduled Bromocriptine or low-dose nonselective *β*-blocker in high-risk patients.

## References

[1] Blackman JA, Patrick PD, Buck ML, Rust RS (2004). Paroxysmal autonomic instability with dystonia after brain injury. *Archives of Neurology*.

[2] Boeve BF, Wijdicks EFM, Benarroch EE, Schmidt KD (1998). Paroxysmal sympathetic storms (“ diencephalic seizures”) after severe diffuse axonal head injury. *Mayo Clinic Proceedings*.

[3] Penfield W (1929). DIencephalic autonomic epilepsy. *Archives of Neurology and Psychiatry*.

[4] Do D, Sheen VL, Bromfield E (2000). Treatment of paroxysmal sympathetic storm with labetalol. *Journal of Neurology Neurosurgery and Psychiatry*.

[5] Baguley IJ, Nicholls JL, Felmingham KL, Crooks J, Gurka JA, Wade LD (1999). Dysautonomia after traumatic brain injury: a forgotten syndrome?. *Journal of Neurology Neurosurgery and Psychiatry*.

[6] Perkes I, Baguley IJ, Nott MT, Menon DK (2010). A review of paroxysmal sympathetic hyperactivity after acquired brain injury. *Annals of Neurology*.

[7] Bower RS, Sunnarborg R, Rabinstein AA, Wijdicks EFM (2010). Paroxysmal sympathetic hyperactivity after traumatic brain injury. *Neurocritical Care*.

[8] Hendricks HT, Heeren AH, Vos PE (2010). Dysautonomia after severe traumatic brain injury. *European Journal of Neurology*.

[9] Wang VY, Manley G (2008). Recognition of paroxysmal autonomic instability with dystonia (PAID) in a patient with traumatic brain injury. *Journal of Trauma*.

[10] Scott JS, Ockey RR, Holmes GE, Varghese G (1997). Autonomic dysfunction associated with locked-in syndrome in a child. *American Journal of Physical Medicine and Rehabilitation*.

[11] Thorley RR, Wertsch JJ, Klingbeil GE (2001). Acute hypothalamic instability in traumatic brain injury: a case report. *Archives of Physical Medicine and Rehabilitation*.

[12] Macmillan C, Grant I, Andrews P (2002). Pulmonary and cardiac sequelae of subarachnoid haemorrhage: time for active management?. *Intensive Care Medicine*.

[13] Russo RN, O’Flaherty S (2000). Bromocriptine for the management of autonomic dysfunction after severe traumatic brain injury. *Journal of Paediatrics and Child Health*.

[14] Sakr YL, Ghosn I, Vincent JL (2002). Cardiac manifestations after subarachnoid hemorrhage: a systematic review of the literature. *Progress in Cardiovascular Diseases*.

[15] Bhigjee AI, Ames FR (1985). Adult aqueduct stenosis and diencephalic epilepsy. A case report. *Journal of the Neurological Sciences*.

[16] Diamond AL, Callison RC, Shokri J, Cruz-Flores S, Kinsella LJ (2005). Paroxysmal sympathetic storm. *Neurocritical Care*.

[17] Naredi S, Lambert G, Edén E (2000). Increased sympathetic nervous activity in patients with nontraumatic subarachnoid hemorrhage. *Stroke*.

[18] Moussouttas M, Lai EW, Khoury J (2012). Determinants of central sympathetic activation in spontaneous primary subarachnoid hemorrhage. *Neurocritical Care*.

[19] Lee VH, Oh JK, Mulvagh SL, Wijdicks EFM (2006). Mechanisms in neurogenic stress cardiomyopathy after aneurysmal subarachnoid hemorrhage. *Neurocritical Care*.

[20] Bruder N, Rabinstein A (2011). Cardiovascular and pulmonary complications of aneurysmal subarachnoid hemorrhage. *Neurocritical Care*.

[21] De Chazal I, Parham WM, Liopyris P, Wijdicks EFM (2005). Delayed cardiogenic shock and acute lung injury after aneurysmal subarachnoid hemorrhage. *Anesthesia and Analgesia*.

[22] Sheikhazadi A, Gharehdaghi J (2009). Survey of sudden death from aneurysmal subarachnoid hemorrhage in cadavers referred to legal medicine organization of Tehran, 2001–2005. *American Journal of Forensic Medicine and Pathology*.

[23] Schievink WI, Wijdicks EFM, Parisi JE, Piepgras DG, Whisnant JP (1995). Sudden death from aneurysmal subarachnoid hemorrhage. *Neurology*.

[24] Baumann A, Audibert G, McDonnell J, Mertes PM (2007). Neurogenic pulmonary edema. *Acta Anaesthesiologica Scandinavica*.

